# Effect of the Big Five Personality on the intention to use mHealth applications among the Chinese elderly: a national-based study

**DOI:** 10.3389/fpsyg.2025.1479204

**Published:** 2025-01-30

**Authors:** Jinghui Chang, Anqi Li, Xixi Yang, Chunnan Li, Yanshan Mai, Dayi Zhang, Wende Yan, Yibo Wu, Jiangyun Chen

**Affiliations:** ^1^School of Health Management, Southern Medical University, Guangzhou, China; ^2^Department of Global Health and Population, Harvard Chan School of Public Health, Harvard University, Boston, MA, United States; ^3^School of Public Health, Southern Medical University, Guangzhou, China; ^4^School of Public Health, Peking University, Beijing, China

**Keywords:** Big Five Personality, a cross-sectional study, influencing factor, mobile consumer behavior, mobile health (mHealth)

## Abstract

**Background:**

Mobile health applications may provide potential tools to support healthy aging. Promoting mHealth use by the elderly remains an imperative to realize health improvement.

**Objective:**

The research aimed to identify the relationship between personality traits and the intention to use mHealth applications among the elderly in mainland China.

**Methods:**

Using a multi-stage sampling method, a cross-sectional survey of 3,712 older adults across China was obtained out of 3,721 (validity rate was 99.76%) in 2022. The effect of personality on the intention to use mHealth applications among older adults was analyzed by multiple linear regression. The independent variable was the personality, using the short version of the Big Five Personality Inventory (BFI-10). The dependent variable was the intention to use the mHealth applications, measured using a sliding scale. Moderators included demographic characteristics and psycho-social variables measured by the Perceived Social Support Scale, the Family Health Scale, and the Health Literacy Scale.

**Results:**

The Chinese older adults’ intention to use mHealth applications scored (63.31 ± 25.09). Out of the five traits, extraversion, agreeableness, and openness exerted significant effects. Higher scores of extraversion ( β =0.59, t = 1.99, *p* < 0.05) and openness ( β =1.87, *t* = 6.07, *p* < 0.01) contributed to the intention to use mHealth applications. Conversely, higher agreeableness scores ( β = − 1.05, *t* = −3.12, *p* < 0.01) related to lower intention.

**Conclusion:**

The elderly with high extraversion, openness, and low agreeableness are more willing to adopt mHealth applications in China. Healthcare professionals and mHealth device developers can make personalized and accurate interventions for the elderly with different personalities.

## Introduction

In recent years, the Chinese elderly population has grown significantly, leading to a deepening of societal aging. By the end of 2021, there were 264 million people aged 60 and above, accounting for 18.7% of the total population. Of these, more than 180 million older adults suffer from chronic diseases, with the proportion of elderly people having more than one chronic disease as high as 75% (Seventh National Population Census Bulletin in China (No. 5), 2021). China possesses the largest elderly population globally. The substantial proportion of elderly individuals and the demand for disease management and monitoring present an immense economic burden and significant challenge to China. The emergence of mobile health (mHealth) applications offers potentially innovative solutions for managing and achieving healthy aging. mHealth applications were a novel platform that uses mobile applications to acquire data across health conditions, disease diagnosis, prevention, and management (Check Hayden, 2016; Digital Health Center of Excellence, 2022). mHealth applications enable real-time monitoring of users’ health data and behaviors, continuous tracking and recording of user information, provision of more accurate clinical information and data feedback to healthcare professionals, assistance in early disease intervention, assessment, and risk prediction management (Hayes et al., 2014; Shaw et al., 2015). Examples of such applications include devices like ambulatory glucose monitoring devices, Oura Ring, Apple Watch, and Xiaomi Watch. These devices are typically paired with complementary software (e.g., Oura, Apple Health, and Activity) to track and record health-related data. Furthermore, these applications guide users in self-monitoring, self-management, and health behavior improvement, enhancing the efficacy of home healthcare (Birjandtalab et al., 2017; Emanuela et al., 2017). In addition, mHealth applications allow users to assess their health status more accurately through platforms such as online consultations and online healthcare, and to access mobile interventions that aid in disease prevention and treatment, ultimately helping to alleviate pressure on the healthcare system (Adams et al., 2017).

Despite the documented benefits of mHealth applications in disease monitoring and management (Vázquez-de Sebastián et al., 2021), studies suggested that achieving high acceptance and utilization rates among elderly users remains challenging, primarily due to their typically lag behind in technological innovations (Deng et al., 2014; Rogers et al., 2014). Previous studies have identified two key psychological barriers among elderly users in China: technology anxiety and dispositional resistance to change, both of which significantly inhibit their engagement with mobile health services (Guo et al., 2013). The usage to manage their chronic diseases is limited (Matthew-Maich et al., 2016). However, according to the data flow, mHealth apps are gaining in popularity among the Chinese population under the content of the COVID-19 outbreak (Yang et al., 2023). Prior research has suggested that individuals who are young or middle-aged, obese or overweight, possess a high level of education, or have good economic status are more likely to use mHealth applications (Li et al., 2019). Besides examining demographic characteristics that affect users’ adoption of mHealth applications, previous studies have focused on constructing behavioral models of users’ adoption of mobile health services from the perspectives of usage performance (Azeez and Mohammed, 2022), technology perception, technology anxiety, and social impact (Hoque and Sorwar, 2017).

Since the 20th century, researchers have reached a relatively consistent consensus on the personality description models, proposing the Big Five model with stability and universality, which has been increasingly corroborated through studies of broader samples (Terenzini et al., 1981). The Big Five Inventory-10 has 5 dimensions, which are extraversion, agreeableness, conscientiousness, neuroticism, and openness (Rammstedt and John, 2007). According to Costa and McCrae (2011), these 5 personality traits are characterized as follows: individuals high in neuroticism tend to experience emotional instability and negative affect frequently; those with elevated extraversion actively pursue social engagement and are outgoing; people with high openness demonstrate intellectual curiosity and receptiveness to novel experiences; individuals with strong agreeableness exhibit compassion and cooperative tendencies in interpersonal relationships; and those with high conscientiousness display responsibility, and self-discipline behaviors. Several empirical studies from around the world link personality characteristics to the way people use mobile applications and social platforms. According to these studies, extroverted people tend to be more active users of social media (Correa et al., 2010; McGowan et al., 2022), smart wearable applications (Rauschnabel et al., 2015), and health-related digital technologies (Wagner et al., 2017; Huseynov, 2020) than more introverted people. Agreeableness is negatively related to internet usage (Wagner et al., 2017; Huseynov, 2020) and internet addiction (Randler et al., 2014; Kayiş et al., 2016). Studies have revealed that openness is positively related to the utilization of smart wearable technology (Rauschnabel et al., 2015) and social media (Correa et al., 2010) such as health-related applications (Huseynov, 2020). On the basis of the current research, it can be hypothesized that personality traits are associated with the willingness of users to use mHealth applications.

In summary, previous studies involving diverse populations have shown that psychological traits, including personality characteristics, are associated with users’ internet usage, addiction, and interactions with wearable devices, as well as their continued use. However, due to the unique characteristics of older adults, such as their physical health status and behavioral capabilities, significant differences exist when compared to the general population. Existing research on older adults’ use of mobile health devices has focused on rational aspects such as application design (Kruse et al., 2017; Wang Q. et al., 2022), physical age-related limitations, and demographic characteristics (Li et al., 2019). The psychological mechanisms and irrational traits influencing the use of mobile health applications in older adults remain an important area that warrants further investigation. Therefore, this study extends the current understanding by investigating the role of personality traits in elderly users’ mHealth acceptance. Consequently, this study analyzed the relationship between Chinese elderly’s personality traits and willingness to adopt mHealth applications from a questionnaire-based cross-sectional survey, which was conducted by Psychology and Behavior Investigation of Chinese Residents, PBICR, in 2022. This study aims to contribute to enhancing the benefits of using mHealth applications for older adults, addressing the issue of population aging, and achieving healthy aging.

## Methods

### Study design and setting

The data for this study were sourced from the PBICR survey. A questionnaire-based cross-sectional survey was conducted in 2022 in 23 provinces, 5 autonomous regions, and 4 municipalities directly under the central government in mainland China. Multistage sampling was used in districts, counties, and communities. The survey is based on quota attributes (ie, sex, age, and urban–rural distribution) of China’s seventh national census data. The protocol provided detailed sampling methods (Wang Y. et al., 2022).

The survey procedure was as follows: During the survey period, posters were displayed and paper or electronic recruitment notices were distributed by the investigator to attract respondents. The investigators adopted the face-to-face survey but distributed the electronic questionnaire made by the online tool (named “Questionnaire Star”) in the communities on the spot, which can be obtained by scanning the QR code. The investigator verified the identity of the respondents and determined that the respondents checked the inclusion and exclusion criteria. Then the participants finished the questionnaire with the accompany of the investigator in case they have any questions. If the respondent has the ability to think but is not mobile enough to answer the questionnaire, the investigator conducts a one-on-one interview and answers on their behalf. If the face-to-face survey cannot be carried out due to the COVID-19 epidemic, the investigators distributed electronic questionnaires to the respondents via video and conducted online video surveys using video chatting software.

**Figure 1:**
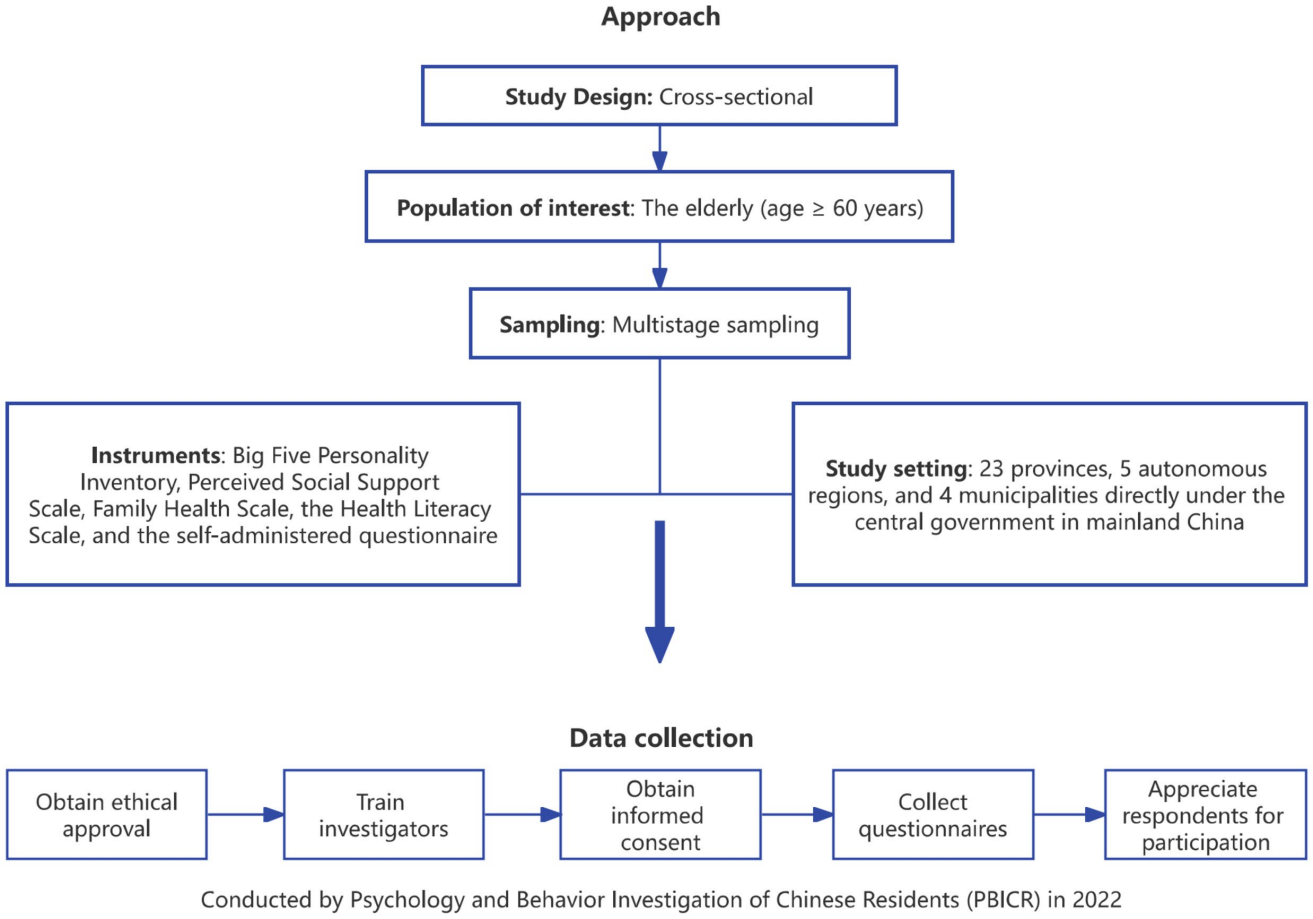
Schematic representation of the research approach.

This study has gone through more than 30 expert consultations and 3 rounds of pre-investigation. This study was registered in the China Clinical Trial Registry (ChiCTR2200061046) (15/06/2022). The study approach is presented in Figure 1.

### Participants

A total of all-age 21,916 questionnaires were collected and prepared for screening for the elderly (aged 60 and over). From the all-age 21,916 samples, 3,721 questionnaires were screened under the condition “aged 60 and above.” Out of 3,721, missing data and logical errors were excluded, which resulted in 3712 valid data (the validity rate was 99.76%).

The inclusion criteria for this study were as follows: (1) Aged 60 and above; (2) Had the nationality of the People’s Republic of China; (3) Chinese permanent resident population with an annual travel time ≤ 1 month; (4) Participated in the study and fill in the informed consent form voluntarily; (5) Participants can complete the questionnaire survey by themselves or with the help of investigators; (6) Participants can understand the meaning of each item in the questionnaire. Exclusion criteria were the following: (1) Persons with mental disorders; (2) Persons with cognitive impairment; (3) Those who were participating in other similar research projects; (4) Reluctant collaborators.

### Measurements

#### The Big Five Personality

The 10-Item Short Version of the Big Five Inventory is an abbreviated version of the BFI developed by Rammstedt and John and retains a high degree of reliability (Marcus et al., 2012). It has been widely used in personality measurement and research for many years as it retains a high reliability and validity. But also measures personality traits more efficiently and conveniently. The Big Five Personality scale includes extraversion, agreeableness, conscientiousness, neuroticism, and openness, and uses a 5-point Likert scale to rate 10 items from 1 (strongly disagree) to 5 (strongly agree). Scores for extraversion were added to items 1R and 6, scores for agreeableness were combined with scores for items 2 and 7R, scores for conscientiousness were 3R and 8, scores for neuroticism were 4R + 9, and scores for openness were 5R + 10 (R = items as reverse scores). Higher scores indicate higher levels of that personality trait. The study confirmed that the BFI-10 has psychometric properties comparable to those of the full-scale BFI (Carciofo et al., 2016).

#### The intention to adopt mobile health applications

Using a sliding scale of 0–100 to measure intention to adopt mHealth applications in a electronic questionnaire on the phone, participants were asked to drag the sliding scale to rate their intention to adopt mHealth applications. mHealth applications were explained as a novel platform that uses mobile applications to acquire data across health conditions and disease diagnosis, prevention, and management (Check Hayden, 2016). 0 being absolutely not willing to use mHealth applications and 100 being very acceptable.

#### Moderators

Moderators, according to previous studies, were identified based on their association with the independent variable (Big Five Personality) and their impact on changes in the association between the independent and dependent variables (mHealth applications adoption intentions). Respondents’ socio-demographic characteristics were used as moderators in the analyses for the general sample, including religion, politics, education, monthly income, body mass index (BMI, calculated by reported height and weight), residence, and chronic diseases (self-reported). Other moderators would be included as potential confounders in the final models if they affected the associations between the Big Five Personality and intended adoption. The potential confounders are as follows.

Family health is measured using the Chinese Short Form of the Family Health Scale (FHS-SF) developed by Crandall and Weiss. FHS-SF assesses family health function using 10 items (Crandall et al., 2020). Each question was rated on a 5-point Likert scale from 1 (strongly disagree) to 5 (strongly agree) for the item, with questions 6, 9, and 10 using a reverse scale. Higher scores are associated with better family health. Cronbach’s alpha for the FHS-SF was 0.80.

The Perceived Social Support Scale (PSSS), based on Zimet et al., is a 12-item scale that assesses the perceived level of emotional support from social relationships (Zimet et al., 1990; Li et al., 2020). It is scored from 1 to 7 (Likert) according to the options “very strongly disagree” to “very strongly agree.” The total score for the summation items ranged from 12 to 84, with higher scores indicating a higher perception of social support. Coefficient alpha values ranged from 0.84 to 0.92 for the scale as a whole.

**Figure 2:**
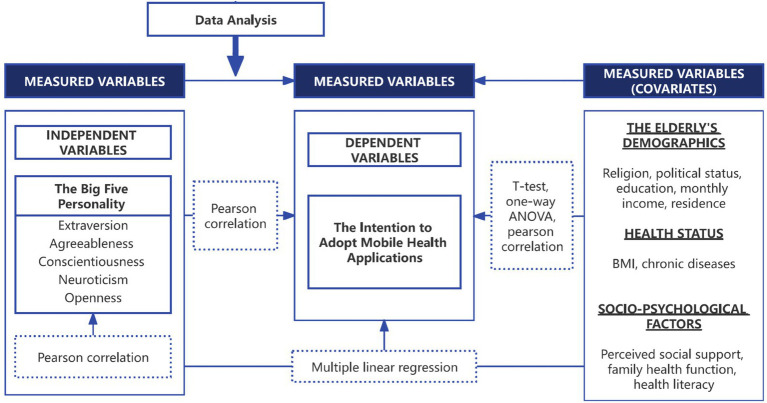
Variables to be analyzed in this study.

The Health Literacy Scale-Short Form-9 is a scale based on the Health Literacy Scale-Short Form-12, a measure of health literacy developed by Duong et al. It is divided into 4 dimensions and 9 questions that measure the ability to find, understand, evaluate, and apply health-related information (Duong et al., 2019). Items are scored on a 4-point Likert scale ranging from 0 ‘very difficult’ to 3 ‘very easy’, with summed scores ranging from 0 to 27, with higher scores indicating higher levels of health literacy. Cronbach’s alpha of the Short Form-12 was 0.85. Variables included in the study are presented in Figure 2.

### Statistical analysis

Data entry and analysis were performed using SPSS (version 27.0). Categorical variables are presented as the number of participants and percentage. First, the Kolmogorov–Smirnov test was used to determine whether continuous variables were normal. Categorical variables are presented as the mean and standard deviation and student’s t-test and one-way ANOVA were used to compare differential factors for the adoption intention score among the Chinese older adults. Pearson correlation was used to analyze the correlation among the continuous variables.

The multiple linear regression analysis was applied to estimate factors associated with the adoption intention score among Chinese elderly residents. The score of the adoption intention was taken as the dependent variable. The independent variables mainly included socio-demographic characteristics and psychological information, among which the unordered multiple categorical variables and ordered multiple categorical variables were converted into multiple dummy variables. A *p*-value less than 0.05 was considered significant. The regression coefficients (*β*) and respective 95%CIs were reported.

## Results

### The statistical descriptive

The demographic information and statistical description of the participants are presented in Table 1. First, the Kolmogorov–Smirnov test showed that the score of willingness to adopt mHealth applications was normal (*p* = 0.07). A total of 3,712 participants were recruited for the study, 49.5% of whom were male. The average age of the participants was (69.23 ± 6.13) years old, over 90% of the elderly were Chinese Han nationality and had no religious beliefs. Only 12.6% of the elderly had a college education or higher. 13.8% of participants were members of Communist Party of China. Over 60% of the elderly had an income of less than 4,000 CNY per month. 43.6% of participants lived in rural areas for the past 3 months. The household registration of 60.8% of participants is rural. In terms of health status, 63.4% of the elderly had a normal BMI and more than half of the elderly had chronic diseases. The mean scores of other scales were as follows: PSSS (14.47 ± 3.24), FHS (37.85 ± 6.45), and HLS (37.85 ± 6.45).

**Table 1:** Participant demographics and comparison of elderly people’s adoption intention to mHealth applications (*n* = 3,712).

Variables	Groups	*n* (%)	The mean total score of adoption intention	F/t/r	*p*
Total		3,712 (100.00)	63.31 ± 25.09		
Sex				*t* = 0.71	0.48
	Male	1839 (49.54)	63.61 ± 25.30		
	Female	1873 (50.56)	63.02 ± 24.88		
Age group				*F* = 1.65	0.19
	60 ~ 74	3,036 (81.79)	63.57 ± 24.76		
	75 ~ 89	662 (17.83)	62.31 ± 26.33		
	≥ 90	14 (0.38)	54.00 ± 32.62		
Nationality				*t* = −1.96	0.05
	Han nationality	3,370 (90.79)	63.95 ± 25.16		
	Others	342 (9.21)	65.85 ± 24.21		
Religion				*t* = 2.21	0.03
	No	3,416 (92.03)	63.58 ± 25.14		
	Yes	296 (7.97)	60.22 ± 24.32		
Political status				*F* = 11.13	<0.01
	General public	3,151 (84.89)	62.56 ± 25.02		
	Member of the Communist Party of China	512 (13.79)	68.14 ± 25.08		
	Others	49 (1.32)	61.29 ± 27.18		
Education				*F* = 18.21	<0.01
	Below elementary school	1817 (48.95)	61.41 ± 25.76		
	Junior high school to senior high school	1,426 (38.42)	63.83 ± 24.58		
	College or above	469 (12.63)	69.11 ± 22.93		
Monthly income (CNY^a^¥ 1 [US $1.147])				*F* = 14.45	<0.01
	≤ 2000	910 (24.52)	59.29 ± 25.57		
	2001 ~ 4,000	1,410 (37.98)	62.91 ± 25.28		
	4,001 ~ 6,000	830 (22.36)	65.00 ± 22.72		
	6,001 ~ 12,000	453 (12.20)	66.94 ± 21.99		
	> 12,000	109 (2.94)	74.06 ± 23.86		
Residence for 3 months				*t* = −4.26	<0.01
	Rural area	1,620 (43.64)	62.32 ± 25.43		
	City	2092 (56.36)	64.85 ± 24.71		
Registered permanent residence				*t* = −4.98	<0.01
	Agricultural	2,258 (60.83)	61.67 ± 25.26		
	Non-agricultural	1,454 (39.17)	65.86 ± 24.61		
BMI Rank				*F* = 6.53	<0.01
	Normal(18.5 ~ 23.9)	2,355(63.44)	63.92 ± 24.50		
	Underweight( < 18.5)	373(10.05)	58.68 ± 26.98		
	Overweight(24 ~ 27.9)	841(22.66)	64.36 ± 25.48		
	Obese( ≥ 28)	143(3.85)	59.17 ± 25.68		
Chronic diseases				*t* = 5.95	<0.01
	No	1,552 (41.81)	66.19 ± 24.41		
	Yes	2,160 (58.19)	61.24 ± 25.37		
Home style				*F* = 1.17	0.11
	Couples families	887 (23.90)	63.55 ± 25.30		
	Nuclear families	325 (8.76)	64.00 ± 25.61		
	Main families	1854 (49.95)	63.32 ± 24.35		
	Others	646 (17.40)	62.60 ± 26.61		
PSSS scores		3,712 (100.00)	63.31 ± 25.09	*r* = 0.16	<0.01
FHS scores		3,712 (100.00)	63.31 ± 25.09	*r* = 0.08	<0.01
HLS scores		3,712 (100.00)	63.31 ± 25.09	*r* = 0.20	<0.01

^aCNY: Chinese Yuan.^

Different demographic groups showed a significant difference in intention to use mHealth applications among the Chinese elderly, as well as other psychological indicators, including religion(*t* = 2.21, *p* = 0.03), political status(*F* = 11.13, *p* < 0.01), education(*F* = 18.21, *p* < 0.01), monthly income(*F* = 14.45, *p* < 0.01), Residence for 3 months(*t* = −4.26, *p* < 0.01), registered permanent residence(*t* = −4.98, *p* < 0.01), BMI rank(*F* = 6.53, *p* < 0.01), chronic disease(*t* = 5.95, *p* < 0.01), PSSS score(*r* = 0.16, *p* < 0.01), FHS score(*r* = 0.08, *p* < 0.01), HLS score(*r* = 0.20, *p* < 0.01).

### The Big Five Personality and adoption intention of mHealth applications

The score of the Big Five Personality is presented in detail (Appendix Table 1). The following were the mean scores of each personality characteristic on the BFI-10 scale: the score of extraversion was (6.14 ± 1.44), the score of agreeableness was (6.90 ± 1.41), the score of conscientiousness was (7.06 ± 1.52), the score of neuroticism was (5.50 ± 1.43) and the score of openness was (5.93 ± 1.33).

**Figure 3:**
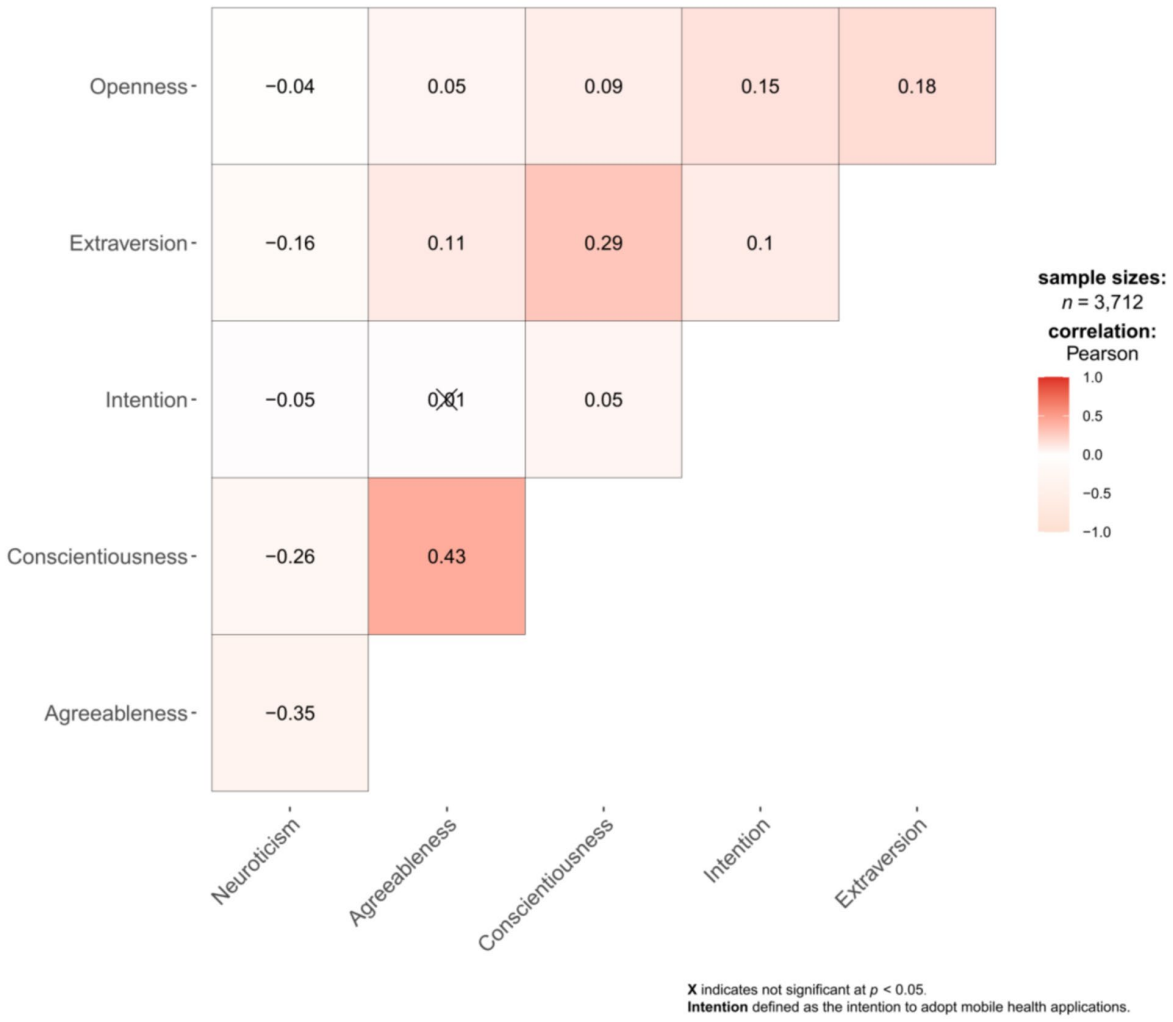
Correlation plot of 5 personality traits and the intention to adopt mHealth applications.

Pearson correlation analysis showed that the absolute values of the correlation coefficients (*r*) among the five personality traits and the intention to adopt mHealth applications ranged from 0.038 to 0.427 (Appendix Table 2). Figure 3 presents the correlation between personality traits and the intention to adopt mHealth applications.

### The factors relevant to the adoption intention of mHealth applications among the elderly

Among all influencing factors, some personality traits were significantly associated with the intention to use mHealth applications. According to linear regression models before adjustment, after adjustment for religion, politics, education, monthly income, BMI, and chronic diseases (adjusted model 1), and after adjustment for all the above factors together with PSSS score, FHS score, and HLS score (adjusted model 2). The results of the multiple linear regression (adjusted model 2) indicated only slight collinearity, with VIF values ranging from 1.028 to 2.014 (all VIFs <5) (Appendix Table 3).

In adjusted model 2, significant moderators are as follows. Chinese older adults with educational attainment of Junior high school to senior high school ( β = − 1.93, *t* = −2.09, *p* = 0.04), underweight ( β = − 4.40, *t* = −3.26, *p* < 0.01) or obese ( β = − 5.97, *t* = −2.85, *p* < 0.01) and suffering from chronic diseases ( β = − 3.07, *t* = −3.72, *p* < 0.01) are less willing to use mHealth applications. However, for the elderly with political status as the members of the Communist Party of China ( β =3.04, *t* = 2.45, *p* = 0.01), monthly income is 4,001 ~ 6,000 CNY ( β =3.82, *t* = 3.09, *p* < 0.01), 6,001 ~ 12,000 CNY ( β =4.78, *t* = 3.25, *p* < 0.01), and 12,000 CNY above ( β =8.20, *t* = 3.21, *p* < 0.01) are more willing to use mHealth applications. Among continuous variables, PSSS score ( β =1.05, *t* = 6.47, *p* < 0.01) and HLS score ( β =0.63, *t* = 7.05, *p* < 0.01) are positively correlated with the willingness to use mHealth applications among the elderly in China, whereas FHS score ( β = − 0.18, *t* = −2.06, *p* = 0.04) is negatively correlated (Appendix Table 3).

As presented in adjusted model 2 ( $R^{2}$ =0.09), three dimensions of personal traits remained statistically significant (*p* < 0.05) after adjusting for demographic variables and health status (political status, monthly income, religion, BMI, chronic diseases) and continuous psychological indicators (PSSS score, HLS score, FHS score). Specifically, extraversion, agreeableness, and openness exerted statistically significant effects on the adoption intention of mHealth applications among elderly residents (*p* < 0.05). Extraversion ( β =0.59, *t* = 1.99, *p* < 0.05) and openness ( β =1.87, *t* = 6.07, *p* < 0.01) have a significant positive effect on adoption intention whereas agreeableness ( β = − 1.05, *t* = −3.12, *p* < 0.01) plays a negative influencing factor. Agreeableness and openness were the most significant predictors of the Chinese elderly willingness to adopt mHealth applications (Table 2).

**Table 2:** Multiple linear regression of personality traits influencing adoption intention among elderly people (*n* = 3,712).

Personality traits	Unadjusted		Adjusted 1^a^		Adjusted 2^b^	
	$\beta \;\left(95\%CI\right)$	*p* value	$\beta \;\left(95\%CI\right)$	*p* value	$\beta \;\left(95\%CI\right)$	*p* value
Extraversion	1.132 (0.541, 1.772)	< 0.001	1.073 (0.487, 1.659)	< 0.001	0.593 (0.007,1.179)	0.047
Agreeableness	−0.574 (−1.223, 0.075)	0.083	−0.573 (−1.218, 0.072)	0.081	−1.052 (−1.713, −0.390)	0.002
Conscientiousness	0.437 (−0.166, 1.040)	0.156	0.589 (−0.013, 1.190)	0.055	0.097 (−0.519, 0.714)	0.757
Neuroticism	−0.663 (−1.265, −0.060)	0.031	−0.636 (−1.234, −0.038)	0.037	−0.294 (−0.885, 0.298)	0.331
Openness	2.543 (1.935, 3.150)	< 0.001	2.021 (1.408, 2.633)	< 0.001	1.874 (1.269, 2.479)	< 0.001

^aAdjusted 1: adjusting for religion, political status, education, monthly income, BMI, chronic diseases, residence for 3 months, registered permanent residence.^

^bAdjusted 2: adjusting for religion, political status, education, monthly income, BMI, chronic diseases, residence for 3 months, registered permanent residence, PSSS scores, FHS scores, and HLS scores.^

## Discussion

### Principal results

According to the National Informationization Development Evaluation Report, China’s comprehensive level of computing infrastructure ranks second in the world, and its information infrastructure continues to improve (Hao, 2023). Against both informatization and population aging, information technology is becoming a potential measure to support healthy aging and mobile health. Moreover, the driving force of the elderly in the digital economy market should not be underestimated. This study found that, in addition to significant moderators (education, monthly income, political status, BMI, chronic diseases, PSSS score, HLS score, FHS score), three dimensions of Big Five personality traits influence the willingness to use mHealth applications, which is following our theoretical assumption. In detail, high extraversion, low agreeableness, and high openness were associated with a higher willingness to adopt mHealth applications among older adults. Conscientiousness and neuroticism appear insignificantly to their intention.

Among the covariates examined, significant differences in willingness to adopt mHealth applications were observed across various demographic and psycho-social factors among Chinese elderly participants. Specifically, elderly individuals with higher PSSS scores, better HLS scores, members of the Communist Party of China, and higher monthly income demonstrated greater willingness to use mHealth applications. In contrast, those with higher FHS scores, higher education levels, BMI rank of underweight or obese, and those with chronic diseases showed lower willingness to adopt mHealth applications. Prior research has explored the role of family influences on elderly individuals’ access to mHealth services (Jen and Hung, 2010), which indicates the differential role of family among the elderly supported by our findings regarding the role of FHS scores. Notably, our results also reveal that elderly individuals with poorer health status (indicated by BMI ranks of underweight or obese, and chronic diseases) demonstrated lower willingness to adopt mHealth applications. This finding suggests that healthcare providers and mHealth developers should direct additional attention and resources toward these vulnerable populations to enhance their engagement with mHealth technologies.

### Extraversion

A positive correlation between extraversion and older adults’ intention to use mHealth applications. This means that older people with higher levels of extraversion are more likely to adopt mHealth applications. This study aligns with the research conducted by Correa et al. (2010), which demonstrated that extraversion and openness are positively associated with social media usage. Additionally, the present study’s results are congruent with those of McGowan et al. (2022) established that extraversion is a significant positive determinant of perceived persuasiveness in mobile health. Extraverts are characterized by their sociability, energy, and talkativeness (McCrae and John, 1992; MrCrae and Costa, 1999), primarily focus on external stimuli and derive gratification from interpersonal interactions. Consequently, they often maintain extensive social networks and engage in numerous social groups (MrCrae and Costa, 1999). The propensity of highly extraverted individuals to utilize mHealth applications can be attributed to the enhancement of their social experiences, as these applications facilitate online communication, enable the sharing of health-related content on social media platforms, support participation in virtual health courses, and accommodate the use of wearable health applications for outdoor activities. Nevertheless, the current research diverges from previous studies investigating the relationship between personality traits and internet usage. Landers and Lounsbury (2006), for instance, suggested that introverts exhibit higher levels of internet utilization. This discrepancy may be ascribed to the fact that social media applications are less anonymous (Correa et al., 2010) and more socializing than in earlier years. Furthermore, a lot of mHealth applications are designed for effortless remote monitoring, rendering them particularly suitable for health monitoring among extraverted individuals, irrespective of their geographic location. Hence, when considering mHealth applications, extroverted senior citizens exhibit a greater likelihood of engaging with these products. In practical terms, mHealth device developers ought to thoroughly comprehend the requirements of extraverted older adults and cater to their specific preferences by offering personalized products and software solutions. In their professional capacities, healthcare practitioners and administrators of health services are well-positioned to promote and improve the implementation of mHealth applications tailored to extroverted older individuals. Given that extroverts characteristically maintain expansive social networks, leveraging the influence of extroverted senior “opinion leaders” can be advantageous in augmenting the adoption rate of such applications and facilitating the evidence-based management of geriatric health.

### Openness

Openness is positively correlated with Chinese older adults’ propensity to utilize mHealth applications. Older adults who are more open are more likely to employ mHealth applications. The present study’s findings lend support to this result. This outcome is congruent with Huseynov (2020) finding that individuals exhibiting openness to experience tend to allocate more time to health and lifestyle applications. Another investigation underscored the significance of openness, revealing that elevated levels of both openness and extraversion are associated with increased online engagement (Correa et al., 2010). Prensky (2001) observed that seniors with high degrees of openness demonstrated a stronger inclination to use social media compared to their younger counterparts. High levels of openness to experience are typified by curiosity and a penchant for novelty, whereas low levels are characterized by a preference for adherence to convention and established patterns (John and Srivastava, 1999). Given the openness personality trait and previous research, it is not surprising and logical to expect that older people with higher openness would be more likely to use mHealth applications, considering the relative newness and nascent nature of this technology. However, this finding suggests that managers might confront a situation in which, once the technology matures and becomes widely adopted, elderly individuals who exhibit a proclivity for novelty and trend-following could lose their motivation to use mHealth applications. Further investigation is warranted to explore the factors that drive older adults with open personalities to continue utilizing mHealth applications.

### Agreeableness

Agreeableness is negatively related to older adults’ intention to use mHealth applications, and older adults with low agreeableness are more likely to employ mHealth applications. The research by Huseynov’s substantiates the notion that agreeableness is negatively associated with the daily duration and frequency of accessing health and lifestyle-related mobile applications (Huseynov, 2020). Moreover, Kayiş et al. (2016) posited that high levels of openness, conscientiousness, extraversion, and agreeableness serve as protective factors against internet addiction. Agreeableness is characterized by flexibility, courtesy, trustworthiness, cooperation, and tolerance and agreeable individuals are predisposed to forgive, assist, and trust others, as they primarily focus on interpersonal relationships (Norman, 1963; Barrick and Mount, 1991; MrCrae and Costa, 1999). It has been demonstrated that agreeable people strive to avoid internet addiction and generally prefer to engage in direct communication and maintain relationships rather than interact with electronic applications. One study suggested that the avoidance of electronic health technology by agreeable senior citizens can be attributed to older individuals’ lower comprehension and utilization of e-health technology compared to younger people (Jaana and Paré, 2020). Considering previous research, it can be surmised that the primary reason for the reduced inclination of highly agreeable elderly individuals to use mHealth applications is their preference for interpersonal communication over human-computer interaction. In light of the characteristics of highly agreeable older adults, mHealth device developers may prioritize assistive functions, such as focusing on smart wearable devices, without necessitating extensive time and energy expenditure on online social interactions by highly agreeable elderly users. Rupp et al. (2018) also demonstrated that agreeableness indirectly and positively influences the desire and perceived motivational concerns related to wearable applications. Simultaneously, managers can encourage those in the social networks of highly agreeable older individuals, such as family and neighborhood, to adopt mHealth applications collectively. This approach could foster shared conversational topics among highly agreeable older adults and their acquaintances, thereby increasing their willingness to use mHealth applications.

### Conscientiousness

There is no correlation between conscientiousness and older people’s willingness to use mHealth applications according to the result, although some research has suggested that this dimension is related to online business-related applications (Huseynov, 2020). Another previous study showed that Facebook users tend to be more extravert and less conscientious than non-users (Ryan and Xenos, 2011). Certain studies have revealed that conscientiousness served as a protective factor against Internet addiction (Landers and Lounsbury, 2006; Kayiş et al., 2016). While other research has found no significant association between conscientiousness and social media usage (Correa et al., 2010). Ryan and Xenos (2011) propose that the classification of personality traits should be refined, such as narcissism (Mehdizadeh, 2010). Investigations on the use of social platforms also provide insights into the readiness to adopt mHealth applications. Future research should aim to further verify and discuss the consistency and willingness of older adults to use mHealth applications.

### Neuroticism

The result of this study indicates no correlation between neuroticism and older adults’ intention to adopt mHealth applications. The research by Ehrenberg, Jukes, White, and Walsh indicates that people with high neuroticism like to use information software and instant messaging (Ehrenberg et al., 2008). The researchers pointed out that people with high stress are more inclined to use networking software than offline interactions because online interactions give them time to think and process messages, thus reducing the tension and anxiety of communicating with others. Regarding research in the field of smart wearable applications, the study by Rauschnabel et al. (2015) shows that Neurotics tend to be particularly averse to smart glasses if they believe that this technology has a strong impact on their lives. People with high neuroticism oppose smart glasses because they believe that this technology has a strong impact on their lives. In this study, mHealth applications include not only social interaction but also other ways, such as human-computer interaction, which is an application that focuses on supporting and maintaining the original level of health. In other words, the mHealth device does not have social interaction as its main function, and it will not have a great impact on the daily lives of the users. Therefore, this study is not related to the intention of older people to adopt mHealth applications. It does not contradict previous studies, and a more detailed discussion of mHealth applications is needed in the future.

This study provides a basis for how different personality traits of older adults have an impact on the intention to adopt mHealth applications. According to the different personality traits of the elderly, the results of this study show that we can know to what extent who will be willing to adopt and use health-related mobile applications. The above research results can be used by health managers and mHealth applications developers to more accurately grasp the usage tendency of the elderly, strengthen and market their health applications, and promote innovation according to the needs of the elderly, so as to improve the usage rate of mHealth applications for the elderly.

### Limitations

Despite the insights our study offers regarding the intention to adopt and various personality traits in mHealth applications among older adults, certain limitations exist. First, the willingness to use is a self-reported measure that may be subject to measurement and reporting biases. Future studies could be designed to objectively monitor older adults’ usage of mHealth applications. Second, although the Big Five framework is widely employed for assessing personality traits, various other questionnaires and measures are available. In addition, the study collected the demographic and psychological variables, and the measurement of IT literacy should be considered a covariate. Despite these limitations, this study contributes to the research field of older individuals’ intention to use mHealth applications and serves as a reference for future research, mHealth device designers, and managers.

## Conclusion

The findings of this paper are anticipated to contribute to the development of psychological theory regarding the intention to use mHealth applications. In this study, the influence of personality traits on the intention to adopt mHealth applications has been investigated. Personalities were determined utilizing the Big Five personality traits taxonomy, which encompasses extraversion, agreeableness, conscientiousness, neuroticism, and openness. The Mini-IPIP scale was employed to measure the Big Five personality traits among the elderly, while the willingness to use mHealth applications was assessed using a slider to collect data from participants. Featuring a large sample size and broad coverage, this study has focused on older individuals’ intention to adopt mHealth applications. Previous research did not encompass the categories of health-related applications and did not specifically target the elderly population. Considering the frailty of older adults, the high prevalence of chronic diseases, and the pressing issue of active aging in China, employing mHealth applications represents a suitable means of adapting to scientific and technological advancements. This investigation supplements related research on older Chinese individuals’ willingness to use mHealth applications, in addition to traditional influencing factors such as socio-demographic characteristics and psychological indicators.

The study revealed that personality differences play a substantial role in determining the extent to which older individuals are willing to access and utilize mHealth applications. Extraversion and openness facilitated older people’s intentions, whereas agreeableness was detrimental. The two insignificant dimensions, conscientiousness, and neuroticism, warrant further research and validation. Future studies should refine the categories of mHealth applications to enhance research accuracy. Personality traits are also related to various demographic characteristics, such as sex and age, which require additional exploration. Consequently, older adults with high personality traits should be the primary focus of developers and managers. By considering the needs and characteristics of elderly individuals with a low intention to adopt mHealth applications, research and innovation in health applications can potentially improve the usage rate of mHealth applications. This study suggests that mHealth application publishers should take into account the interplay between personality traits and usage behavior when designing and marketing their health applications.

## Data Availability

The raw data supporting the conclusions of this article will be made available by the authors, without undue reservation.
