# Supplementary material for: Effect of the Big Five Personality on the intention to use mHealth applications among the Chinese elderly: a national-based study
Source: Front Psychol. 2025 Jan 30;16:1479204. doi: 10.3389/fpsyg.2025.1479204 (PMC11821963; doi:10.3389/fpsyg.2025.1479204)
Supplement: Supplementary file 1 [file Table_1.docx]

**Appendix Table 1.**Big five personality score of 3712 participants.

| Variables | Items | Mean±SD |
| --- | --- | --- |
| Extraversion |  | 6.14±1.44 |
|  | I am reserved | 2.76±1.01 |
|  | I am outgoing, sociable | 3.38±0.98 |
| Agreeableness |  | 6.90±1.41 |
|  | I am generally trusting | 3.51±0.91 |
|  | I tend to find fault with others | 3.40±1.02 |
| Conscientiousness |  | 7.06±1.52 |
|  | I tend to be lazy | 3.54±1.07 |
|  | I do a thorough job | 3.52±0.94 |
| Neuroticism |  | 5.50±1.43 |
|  | I am relaxed, handle stress well | 2.68±0.96 |
|  | I get nervous easily | 2.82±1.02 |
| Openness |  | 5.93±1.33 |
|  | I have few artistic interests | 2.81±1.06 |
|  | I have an active imagination | 3.11±0.97 |

**Appendix Table 2.**Correlation coefficients (r) of 5 personality traits and the intention to adopt mHealth applications (n=3712).

| Correlations | mHealth adoption intention^a^, *r* (*P* value) | Extraversion, *r* (*P* value) | Agreeableness, *r* (*P* value) | Conscientiousness, *r* (*P* value) | Neuroticism, *r* (*P* value) | Openness, *r* (*P* value) |
| --- | --- | --- | --- | --- | --- | --- |
| mHealth adoption intention | N/A^b^ | N/A | N/A | N/A | N/A | N/A |
| Extraversion | 0.100(＜0.001) | N/A | N/A | N/A | N/A | N/A |
| Agreeableness | 0.006(0.732) | 0.108 (<0.001) | N/A | N/A | N/A | N/A |
| Conscientiousness | 0.053(0.001) | 0.288 (<0.001) | 0.427 (<0.001) | N/A | N/A | N/A |
| Neuroticism | -0.049(0.003) | -0.162 (<0.001) | -0.351 (<0.001) | -0.257 (<0.001) | N/A | N/A |
| Openness | 0.149(＜0.001) | 0.184 (<0.001) | 0.047 (0.009) | 0.085 (<0.001) | -0.038 (0.031) | N/A |

^a^mHealth Adoption Intention defined as the intention to adopt mobile health applications.

^b^N/A: not applicable.

**Appendix Table 3.** Multiple linear regression of factors influencing adoption intention among elderly people after adjusted 2 (n=3712).

| **Variables** | | **Unstandardized coefficient** | | **t** | **P value** | **VIF** |
| --- | --- | --- | --- | --- | --- | --- |
|  |  | **β** | **Standard error** |  |  |  |
| Personality traits |  |  |  |  |  |  |
|  | Extraversion | 0.593 | 0.299 | 1.985 | 0.047* | 1.181 |
|  | Agreeableness | -1.052 | 0.337 | -3.117 | 0.002* | 1.461 |
|  | Conscientiousness | 0.097 | 0.314 | 0.309 | 0.757 | 1.473 |
|  | Neuroticism | -0.294 | 0.302 | -0.973 | 0.331 | 1.197 |
|  | Openness | 1.874 | 0.309 | 6.072 | ＜0.001* | 1.089 |
| PSSS scores |  | 1.052 | 0.163 | 6.468 | ＜0.001* | 1.780 |
| FHS scores |  | -0.179 | 0.087 | -2.063 | 0.039* | 2.014 |
| HLS scores |  | 0.632 | 0.090 | 7.047 | ＜0.001* | 1.466 |
| Religion (Reference: No) |  |  |  |  |  |  |
|  | Yes | -1.618 | 1.477 | -1.096 | 0.273 | 1.028 |
| Political status (Reference: General public) |  |  |  |  |  |  |
|  | Member of the Communist Party of China | 3.043 | 1.243 | 2.449 | 0.014* | 1.180 |
|  | Others | -0.406 | 3.506 | -0.116 | 0.908 | 1.029 |
| Education (Reference: Below elementary school) |  |  |  |  |  |  |
|  | Junior high school to senior high school | -1.928 | 0.922 | -2.091 | 0.037* | 1.293 |
|  | College or above | -0.074 | 1.435 | -0.052 | 0.959 | 1.460 |
| Monthly Income (Reference: $\leq$2000 CNY) |  |  |  |  |  |  |
|  | 2001~4000 CNY | 1.552 | 1.064 | 1.458 | 0.145 | 1.716 |
|  | 4001~6000 CNY | 3.820 | 1.235 | 3.093 | 0.002* | 1.702 |
|  | 6001~12000 CNY | 4.775 | 1.471 | 3.246 | 0.001* | 1.491 |
|  | $>$12000 CNY | 8.199 | 2.552 | 3.213 | 0.001* | 1.193 |
| BMI rank (Reference: Normal) |  |  |  |  |  |  |
|  | Underweight($<$18.5) | -4.400 | 1.348 | -3.263 | 0.001* | 1.057 |
|  | Overweight(24~27.9) | 0.109 | 0.976 | 0.111 | 0.911 | 1.073 |
|  | Obese($\geq$28) | -5.968 | 2.093 | -2.851 | 0.004* | 1.043 |
| Chronic diseases (Reference: No) |  |  |  |  |  |  |
|  | Yes | -3.068 | 0.825 | -3.719 | ＜0.001* | 1.064 |
| Residence for 3 months (Reference: City) |  |  |  |  |  |  |
|  | Rural Area | -0.866 | 1.010 | -0.858 | 0.391 | 1.613 |
| Registered permanent residence (Reference: Non-agricultural) |  |  |  |  |  |  |
|  | Agricultural | 1.385 | 1.022 | 1.356 | 0.175 | 1.598 |

Note: * indicates that the p-value is ＜0.05.
